# Effects of Rising Temperature on the Growth, Stoichiometry, and Palatability of Aquatic Plants

**DOI:** 10.3389/fpls.2018.01947

**Published:** 2019-01-08

**Authors:** Peiyu Zhang, Bart M. C. Grutters, Casper H. A. van Leeuwen, Jun Xu, Antonella Petruzzella, Reinier F. van den Berg, Elisabeth S. Bakker

**Affiliations:** ^1^Department of Aquatic Ecology, Netherlands Institute of Ecology (NIOO-KNAW), Wageningen, Netherlands; ^2^Institute of Hydrobiology, Chinese Academy of Sciences, Wuhan, China

**Keywords:** carbon, climate change, herbivory, macrophyte, nitrogen, nutrient ratio, phosphorus, trophic interaction

## Abstract

Global warming is expected to strengthen herbivore-plant interactions leading to enhanced top-down control of plants. However, latitudinal gradients in plant quality as food for herbivores suggest lower palatability at higher temperatures, but the underlying mechanisms are still unclear. If plant palatability would decline with temperature rise, then this may question the expectation that warming leads to enhanced top-down control. Therefore, experiments that directly test plant palatability and the traits underlying palatability along a temperature gradient are needed. Here we experimentally tested the impact of temperature on aquatic plant growth, plant chemical traits (including stoichiometry) and plant palatability. We cultured three aquatic plant species at three temperatures (15, 20, and 25°C), measured growth parameters, determined chemical traits and performed feeding trial assays using the generalist consumer *Lymnaea stagnalis* (pond snail). We found that rising temperature significantly increased the growth of all three aquatic plants. Plant nitrogen (N) and phosphorus (P) content significantly decreased, and carbon (C):N and C:P stoichiometry increased as temperature increased, for both *Potamogeton lucens* and *Vallisneria spiralis*, but not for *Elodea nuttallii*. By performing the palatability test, we found that rising temperatures significantly decreased plant palatability in *P. lucens*, which could be explained by changes in the underlying chemical plant traits. In contrast, the palatability of *E. nuttallii* and *V. spiralis* was not affected by temperature. Overall, *P. lucens* and *V. spiralis* were always more palatable than *E. nuttallii*. We conclude that warming generally stimulates aquatic plant growth, whereas the effects on chemical plant traits and plant palatability are species-specific. These results suggest that the outcome of the impact of temperature rise on macrophyte stoichiometry and palatability from single-species studies may not be broadly applicable. In contrast, the plant species tested consistently differed in palatability, regardless of temperature, suggesting that palatability may be more strongly linked to species identity than to intraspecific variation in plant stoichiometry.

## Introduction

Global warming is one of the most urgent threats to our ecosystems (IPCC, 2014). The effect of warming has become visible in aquatic ecosystems by rising surface water temperatures and a reduction in ice cover over the last decades (Mooij et al., 2005; Woolway et al., 2017). Temperature rise is furthermore expected to lead to alterations in aquatic communities and their food web interactions (Meerhoff et al., 2012). Several of these changes are already observed: average fish size in temperate fish communities decreases with increasing water temperatures, and the communities tend to contain a higher proportion of omnivorous fishes at the expense of carnivory (Jeppesen et al., 2010). Even without shifting their diet, warming increases the plant consumption rate of plants by ectotherm omnivores and herbivores (Zhang et al., 2018). Warming is thus expected to strengthen herbivore-plant interactions leading to enhanced top-down control of plants (O'Connor, 2009; Gutow et al., 2016).

However, these predictions do not take into account that warming might also affect the plant traits that determine their palatability to herbivores. If plant palatability declines with temperature, then this may alter the expectation that warming leads to enhanced top-down control (O'Connor, 2009). Studies mimicking global warming showed inconsistent effects of temperature on plant palatability, either decreasing palatability in marine plants (Rodil et al., 2015), or having no discernible effect in either terrestrial (Backhaus et al., 2014) or marine plants (Poore et al., 2016). Temperature has been suggested to underlie latitudinal gradients in plant quality as food for herbivores: more palatable plants at higher latitudes suggest lower palatability at higher temperatures (Pennings et al., 2007; Morrison and Hay, 2012). However, the underlying mechanisms are still unclear: this might be because plants are better defended at lower latitudes (Bolser and Hay, 1996), or because plant nitrogen and phosphorus content increases with latitude (Reich and Oleksyn, 2004; Schemske et al., 2009) or both (Grutters et al., 2017). In addition, not all studies find latitudinal effects on plant palatability (Adams et al., 2009; Moles et al., 2011), and factors other than temperature may be causing the observed patterns. Therefore, experiments that directly test the effect of temperature on plant palatability and the traits underlying palatability are needed.

Plant palatability depends largely on three groups of plant traits: plant nutritional traits, plant physical structure and plant secondary metabolites (PSM) (Hay, 1996; Cronin et al., 2002; Elger and Lemoine, 2005). With rising temperature, aquatic plants grow faster (Madsen and Brix, 1997; Short and Neckles, 1999). With increased growth, there could be a nutrient (nitrogen and phosphorus in this study) dilution effect: where these nutrients become limited, the nutrient concentrations in the plant decrease, and if the carbon source is not limited, carbon:nitrogen and carbon:phosphorus stoichiometry increases (Dülger et al., 2017; Velthuis et al., 2017). A decrease in plant nitrogen content and increased carbon:nutrient stoichiometry correspond to reduced consumption rates by herbivores (Sterner and Elser, 2002; Cebrian and Lartigue, 2004; Bakker et al., 2016). Plant physical structure or toughness (Gross and Lombardo, 2018), here represented by leaf dry matter content (Pennings et al., 1998; Elger and Willby, 2003), might also increase with rising temperature, as relatively more carbon accumulates in the plant tissues. PSM, in particular phenolic compounds, are produced by aquatic plants and can act as deterring compounds (Dorenbosch and Bakker, 2011; Grutters et al., 2017). However, the effect of warming on PSM in aquatic plants is unknown.

In this study, we tested the effect of temperature on aquatic plant growth, tissue stoichiometry, plant physical structure and PSM and the consumption rates of a generalist consumer. We performed two sequential experiments in which we first grew three common submerged freshwater vascular plant species at three temperatures (15, 20, and 25°C) under standardized nutrient conditions. We determined the resultant plant growth and plant traits and subsequently performed a second experiment to quantify the feeding rates of a generalist consumer on the plants grown at different temperatures. We hypothesized that rising temperature will (1) increase plant growth, (2) decrease plant nutrient concentration and increase carbon:nutrient stoichiometry, (3) increase toughness, (4) decrease consumption rates.

## Materials and Methods

### Aquatic Plant Growth Experiment

We selected three submerged aquatic plant species: *Elodea nuttallii* (Planch.) St. John, *Vallisneria spiralis* L. and *Potamogeton lucens* L. for the plant growth experiment. These species were chosen because of their wide distribution, representation of different plant genera and high palatability to the pond snail *Lymnaea stagnalis* L. (Elger et al., 2004; Grutters et al., 2017). *E. nuttallii* and *P. lucens* propagules were collected near NIOO-KNAW, Wageningen, The Netherlands. *V. spiralis* seedlings were obtained from the local garden center (Tuincentrum De Oude Tol, Wageningen, The Netherlands). For *E. nuttallii*, 7 cm apical shoots with 2 or 3 branches were chosen. For *V. spiralis*, plants were chosen with a shoot length of 24.2 ± 3.7 cm (mean ± SD, *n* = 45), from which the largest outer leaves were removed. This resulted in plants with 5–7 young leaves in the rosette. For *P. lucens*, lower part of stems with 2 or 3 nodes to sprout new roots and leaves were selected, with a length of 23.7 ± 4.9 cm (mean ± SD, *n* = 45). Even though the heights are different, their initial biomass was very low and similar at the beginning, and the height we chosen can also make sure that the plants are alive. Plant propagules were planted in the pots, first acclimated at 20°C for 2 weeks, and then assigned to their final controlled temperatures. We grew these plants in fifteen temperature-controlled aquaria (90 × 50 × 50 cm, l × w × h) at 15, 20, and 25°C, each with five replicate aquaria. These temperatures are within suitable ranges of these species in nature. In each aquarium, we had three replicates per species following a random design, totaling nine plants per aquarium. Fifteen un-sprouted *P. lucens* propagules were replaced at the beginning of the culturing (one pot in each aquarium).

Temperature was controlled by an automatic control system (Cascade Automation Systems, Ridderkerk, the Netherlands). A mixing pump was placed inside each aquarium to circulate the water, equalize the temperature and increase the CO_2_ concentration. In order to reduce nutrient competition between different plants, each individual was cultured in a separate pot (top diameter 12.5 cm, bottom diameter 11 cm and height 11 cm). Each pot was filled to a depth of 7 cm with pond sediment [Pokon Naturado, Veenendaal, the Netherlands; rich in organic matter, but low in total nitrogen (TN) 8.5 ± 1.1 mg g^−1^ dry soil and total phosphorus (TP) 0.23 ± 0.05 mg g^−1^ (*n* = 3, mean ± SD)], and then covered with a 2 cm layer of sand to reduce nutrient release to the water layer. Each aquarium was filled with tap water (TN, 0.087 ± 0.004 mg L^−1^; TP, 0.013 ± 0.0006 mg L^−1^; *n* = 3, mean ± SD) yielding a water depth of 30 cm. Demineralized water was added weekly to compensate for evaporation. Two great ramshorn snails *Planorbarius corneus* L. (shell diameter of 2.7 ± 0.1 cm, mean ± SD, *n* = 30) were added to each aquarium to control periphyton. They only consumed periphyton, not our plant species and equally consumed periphyton on all plants, as tested in pre-trials. Aquaria were individually illuminated by lamps above each aquarium to reach a 16:8 h day: night cycle and each aquarium was individually wrapped in aluminum foil to prevent light interference among aquaria and to increase light intensity within the aquaria by reflection. The light intensity at the water surface was 47.1 ± 3.2 μmol m^−2^ s^−1^ (a moderate light intensity, mean ± SD, *n* = 7).

Plants were grown in the experiment for 16 weeks from August 16th to December 6th 2015. During the experiment, the plants continued their growing season because they were kept indoors without natural seasonality. Water quality parameters were measured five times during the experiment. Conductivity, pH and dissolved oxygen were checked with a multi–meter (Multi 350i/SET, Germany) in the afternoon of each sampling day. Alkalinity was measured by an auto-titration machine by adding acid (0.1M HCl) until a pH of 4.2 was reached (TIM840 titration manager, Germany). Chlorophyll a (Chl a) was determined by a phytoplankton analyser (PHYTO-PAM, WALZ, Germany). Before harvesting the plants, part of the water was replaced by tap water in some of the 20 and 25°C aquaria to decrease the phytoplankton biomass. Ammonium (NH${}_{4}^{+}$), nitrate (NO${}_{3}^{-}$), nitrite (NO${}_{2}^{-}$) and orthophosphate (PO${}_{4}^{3-}$) were analyzed by an AutoAnalyzer (QuAAtro, Seal Analytical, Fareham, UK) after filtering water samples over GF/F filter (Whatman, Maidstone, UK). The values of the water quality parameters during the experiment are given in Figure S1.

Periphyton was quantified by measuring the dry mass of algae removed from a selected plant surface area of *V. spiralis* at the end of the experiment following Zimba and Hopson (1997). The algae were removed by cutting two pieces of *V. spiralis* leaves (leaf surface area of 23.88 ± 6.65 cm^2^, mean ± SD, *n* = 15), as the leaf area of *V. spiralis* is easy to quantify, shaking them in 30 ml of demineralized water for 30 s, filtering the periphyton onto a pre-weighed GF/F filter (Whatman, Maidstone, UK) and drying the material at 60°C for 48 h. The leaf areas of the cut leaves were measured by first scanning the leaves on a piece of A4 paper, then calculating the surface area with ImageJ (Rasband, 2015). The periphyton data are given in Figure S2.

To investigate how the availability of nutrients to the plants from the sediment in their pots was affected by plant species and temperature, sediment porewater was sampled using rhizons (Rhizosphere, Wageningen, the Netherlands) at the end of the experiment; porewater nutrient concentrations were assumed to be equal among the treatments at the start of the experiment. Total dissolved inorganic nitrogen (TIN: including NH${}_{4}^{+}$, NO${}_{3}^{-}$, NO${}_{2}^{-}$) and PO${}_{4}^{3-}$ concentrations in porewater were determined in one pot per species per aquarium, in total 45 pots were measured. At the end of the experiment, part of the plant tissue in each pot was cut for the feeding trials and the rest was harvested to quantify dry biomass. Shoots and roots were separately cleaned by rinsing with tap water until no visible residue material was attached, including periphyton and sediment particles, and dried in the oven at 60°C for 48 h. Plant relative growth rate was calculated according to the equation: Relative growth rate = (ln W_f_ – ln W_i_)/Days; with W_f_ = final dry mass; W_i_ = initial dry mass. Plant initial dry mass was estimated by drying spare plants which were selected randomly (*n* = 30 for *E. nuttallii, n* = 46 for *V. spiralis*, and *n* = 33 for *P. lucens*) at the beginning of the experiment.

### Snail Feeding Experiment

We selected *L. stagnalis* for our feeding trials, a generalist freshwater mollusk that has often been used in feeding trials (Elger and Barrat-Segretain, 2002, 2004; Grutters et al., 2017), which feeds on a wide variety of vascular aquatic plants (Gaevskaia, 1969), and does select the plants based on their traits (Elger et al., 2004; Gross and Lombardo, 2018). *L. stagnalis* has a holarctic distribution overlapping with the plant species that we study, therefore our study represents the aquatic plant-herbivore interactions as they can be found in the field. Mollusks can have a large impact on aquatic plant abundance in the field (Lodge, 1991; Newman, 1991; Wood et al., 2017). For our feeding experiment, egg clusters from a single population (collected in a pond on the terrain of NIOO-KNAW, 51°59′16.9″N, 5°40′23.5″E) were hatched. After two weeks all the juveniles were transferred to plastic buckets, each filled with 15 liters of groundwater (20°C), and exposed to a 16:8 h day: night cycle. The snails were fed butterhead lettuce 5 days per week. Fish food pellets (Velda, Gold Sticks Basic Food, the Netherlands) and chalk were supplied once a week as food supplements, following Grutters et al. (2017). All water was fully replaced once a week. All snails were grown for 2 months before the feeding trials started. Snails used in the trials had an average shell length of 24.0 ± 1.7 mm and a dry mass (excluding their shell) of 0.17 ± 0.04 g (mean ± SD, *n* = 129).

No-choice feeding trials were carried out to assess whether the temperature at which the plants were grown affected their palatability. The trials followed the standard protocol developed for aquatic snails (Elger and Barrat-Segretain, 2002, 2004; Grutters et al., 2017). In total we used 270 plastic beakers (500 ml), each filled with 375 ml ground water. Prior to the feeding trials, all snails were starved for 48 h following the standard protocol, and visible periphyton was removed from all plant material. The 270 beakers were divided into 135 experimental and 135 paired control beakers. Each experimental beaker received plant material from one plant pot, yielding fifteen replicates per plant species grown at each temperature, with in total 135 beakers containing both one snail and plant fragments. The 135 paired control beakers received plant fragments from the same pot as its experimental counterpart that weighed the same amount, to monitor potential autonomous changes in plant mass for each feeding trail during the 24 h feeding experiment. Snails were offered approximately 0.1 g (wet mass) of apical shoot of *E. nuttallii*, about 0.4 g (wet mass) newly grown *V. spiralis* leaves, and about 0.12 g wet mass for *P. lucens*. For *P. lucens*, leaves lower than the third leave from the top were selected, cut into two equally sized portions with the midrib removed. The amount of plant materials we offered to snails differed among the three species because we offered the maximum amounts according to the consumption per snail for each plant species, as determined in pre-trials. This lowered the measurement errors on small amounts of materials and therefore increased the precision of the measurements. All the plant materials (including the control portion) were cleaned to remove periphyton before being offered to the snails. Beakers were covered with mesh to prevent the snails from escaping. All the trials lasted 24 h and were performed with a 16:8 h day: night cycle at a water temperature of 20°C. We performed all experiments at the same temperature of 20°C to ensure that any potential preference of snails for plants grown at different temperatures was only due to plant quality, and any direct influence of water temperature on snail metabolism and feeding rates was avoided. All feeding trials were randomly divided into two sessions, for logistical reasons. After the feeding trials, snails were first frozen to death at −20°C, and the soft body was separated from its shell, then dried in the oven at 60°C for at least 48 h. The mean snail dry mass without shell was 0.07 ± 0.01 g (mean ± SD, *n* = 129). At the end of the feeding experiment all the remaining plant material was collected and also dried in the oven at 60°C for at least 48 h and weighed.

Plant Relative Consumption Rate (RCR) (mg g^−1^ d^−1^) was calculated following Elger and Barrat-Segretain (2002): RCR = [(C_fd_/C_iw_) ^*^ F_iw_ – F_fd_]/S_d_/1day, in which, C_fd_ is the final dry mass of the paired control plant, C_iw_ is the initial wet mass of the paired control plant, F_iw_ is the initial wet mass of the feeding trial plant, F_fd_ is the final dry mass of the feeding trial plant, and S_d_ is the snail dry mass without shell.

### Plant Chemical Analyses

Plant fragments used as control in the feeding trials were analyzed for their dry matter content and chemical composition. Plant dry matter content was determined as the dry mass divided by the wet mass and expressed as percentage. Each plant sample was ground individually in a 2 ml tube on a Tissuelyser II (QIAGEN, Hilden, Germany). Plant carbon (C) and nitrogen (N) were determined on an elemental auto analyser (FLASH 2000, Thermo Scientific, Waltham, MA, USA). Phosphorus (P) content was determined by incinerating and digesting the sample, and then analyzing the phosphate concentration on an Auto Analyzer (QuAAtro method, Seal Analytical, Fareham, UK). For total phenolics analysis, between 2 and 4 mg of plant material was extracted with 1 ml of 80% ethanol for 10 min at 80°C before adding Sodium dodecyl sulfate solution and FeCl_3_ reagent. The resulting reduction of Fe^3+^ to Fe^2+^ was measured at 510 nm on a spectrophotometer (Synergy HT Microplate Reader, BioTek, Winooski, VT, USA) against a tannic acid calibration curve (Hagerman and Butler, 1989; Smolders et al., 2000). We expressed phenolic content as mg tannic acid equivalents per gram plant dry mass.

### Data Analyses

Data were analyzed in multiple linear mixed-effects models. Dependent variables were the 4 plant growth parameters (Shoot biomass, Root biomass, Relative growth rate and Root:Shoot ratio), 2 nutrient parameters (Porewater TIN and PO${}_{4}^{3-}$ concentration), 9 plant traits (Plant dry matter content, C content, N content, P content, C:N ratio, C:P ratio, N:P ratio, Total phenolics concentration and N:Phenolics ratio) and Relative Consumption Rates by snails. Effects of temperature and differences between plant species were tested by including temperature as continuous variable, species as fixed factor and their interaction. Aquarium was included as a random factor. The significance of included terms was tested by model selection based on AICc values (Burnham and Anderson, 2002; Burnham et al., 2011). We discuss the contributions of all terms included in the top ranking models (ΔAICc < 2.0 from the best model, for model selection see Data Sheet 1). A Tukey *post-hoc* test (package: lsmeans) was applied after each linear mixed-effects model test to compare the difference of the means among the three species (Lenth, 2016). To test effects of temperature on the 16 dependent variables for individual species we used a second set of models, in which we included only temperature as a continuous predictor variable and aquarium as a random factor.

Three snails (2.2%) died during the feeding experiment, one snail per plant species treatment. Three *P. lucens* (each from one of the temperature treatments) did not have enough material for the feeding trials. These data points were excluded from the dataset. Pearson's correlations were used to test for correlations among all the different plant traits in all species simultaneously and separately within each species. For the mixed-modeling we used the package nlme (John and Sanford, 2011; Pinheiro et al., 2016) in R version 3. 4. 1 (R Development Core Team, 2017).

## Results

### Plant Growth and Sediment Nutrients

**Figure 1:**
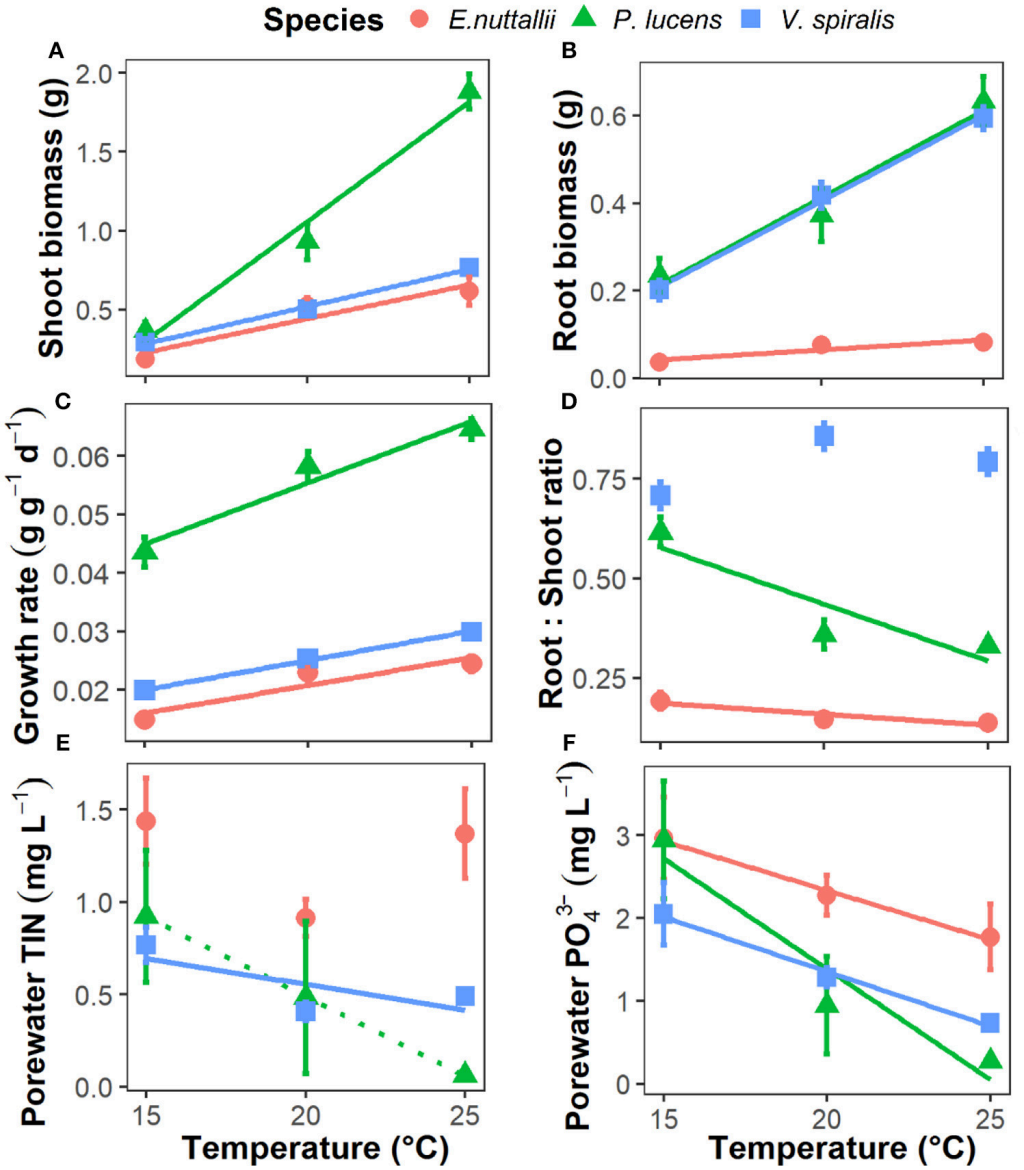
Responses of aquatic plant growth parameters and available nutrients to rising temperature in the three aquatic plants. **(A)** plant shoot biomass, **(B)** plant root biomass, **(C)** plant relative growth rate, **(D)** plant root:shoot ratio, **(E)** sediment pore water total dissolved inorganic nitrogen concentrations (TIN), and **(F)** sediment pore water dissolved inorganic P-PO${}_{4}^{3-}$. Point with bar represents mean ± SE (*n* = 15 for points of growth parameters and *n* = 5 for points of pore water nutrient concentrations). Temperature effects on the parameters of each species are indicated by regression lines. A solid line indicates *p* < 0.05, a dotted line indicates 0.05 < *p* < 0.1, and without line indicates *p* > 0.1.

In our experiment, the final shoot biomass, root biomass, and relative growth rate increased significantly with temperature for all plant species (Table 1, Figures 1A–C). *P. lucens* had the greatest growth, *V. spiralis* intermediate and *E. nuttallii* the least (Table 1, Figures 1A–C). The plant root:shoot ratio showed a species-specific response to increasing temperature: for both *P. lucens* and *E. nuttallii* root:shoot ratios decreased as temperature increased, whereas there was no temperature effect on *V. spiralis*. However, *V. spiralis* had a higher root:shoot ratio than the other two species (Table 1, Figure 1D).

**Table 1:** Linear mixed-effect model results for the effects of temperature and species on the plant growth and sediment pore water nutrient parameters, plant traits and RCR.

**Category**	**Parameters**	**Factors**	**df**	***F***	***p***	**Means comparison among species**
Plant growth	Shoot biomass	Temperature	1, 13	211.93	**<0.001**	*P. lucens* > *V. spiralis* = *E. nuttallii*
		Species	2, 110	82.45	**<0.001**
		Temp.* Species	2, 110	43.68	**<0.001**	
	Root biomass	Temperature	1, 13	56.99	**<0.001**	*P. lucens* = *V. spiralis* > *E. nuttallii*
		Species	2, 110	131.39	**<0.001**	
		Temp.* Species	2, 110	20.87	**<0.001**	
	Growth rate	Temperature	1, 13	43.45	**<0.001**	*P. lucens* > *V. spiralis* > *E. nuttallii*
		Species	2, 110	429.65	**<0.001**	
		Temp.* Species	2, 110	8.47	**<0.001**	
	Root:Shoot ratio	Temperature	1, 13	4.54	0.053	*V. spiralis > P. lucens* > *E. nuttallii*
		Species	2, 110	419.52	**<0.001**	
		Temp.* Species	2, 110	23.27	**<0.001**	
Porewater nutrients	TIN^a^	Temperature	1, 13	3.68	0.077	*E. nuttallii > P. lucens = V. spiralis*
		Species	2, 26	12.62	**<0.001**	
		Temp.* Species	2, 26	2.05	0.149	
	PO${}_{4}^{3-}$	Temperature	1, 13	16.76	**0.001**	*E. nuttallii > P. lucens = V. spiralis*
		Species	2, 26	8.78	**0.001**	
		Temp.* Species	2, 26	3.17	0.059	
Palatability	RCR^b^	Temperature	1, 13	4.01	0.067	*P. lucens* = *V. spiralis* > *E. nuttallii*
		Species	2, 112	8.42	**<0.001**	
Traits	Dry matter content	Temperature	1, 13	2.15	0.166	*P. lucens > E. nuttallii* > *V. spiralis*
		Species	2, 110	448.82	**<0.001**	
		Temp.* Species	2, 110	8.80	**<0.001**	
	C content	Temperature	1, 13	0.83	0.378	*P. lucens > E. nuttallii* = *V. spiralis*
		Species	2, 110	67.57	**<0.001**	
		Temp.* Species	2, 110	16.70	**<0.001**	
	N content	Temperature	1, 13	5.57	**0.035**	*E. nuttallii > P. lucens > V. spiralis*
		Species	2, 110	17.51	**<0.001**	
		Temp.* Species	2, 110	6.85	**0.002**	
	P content	Temperature	1, 13	4.82	**0.047**	*V. spiralis > E. nuttallii > P. lucens*
		Species	2, 110	58.84	**<0.001**	
		Temp.* Species	2, 110	17.33	**<0.001**	
	C:N ratio	Temperature	1, 13	3.02	0.106	*P. lucens* = *V. spiralis* > *E. nuttallii*
		Species	2, 110	9.37	**<0.001**	
		Temp.* Species	2, 110	4.32	**0.016**	
	C:P ratio	Temperature	1, 13	7.14	**0.019**	*P. lucens > E. nuttallii* > *V. spiralis*
		Species	2, 110	68.50	**<0.001**	
		Temp.* Species	2, 110	18.88	**<0.001**	
	N:P ratio	Temperature	1, 13	0.99	0.338	*P. lucens > E. nuttallii* > *V. spiralis*
		Species	2, 110	116.01	**<0.001**	
		Temp.* Species	2, 110	14.64	**<0.001**	
	Total phenolics content	Temperature	1, 13	1.55	0.235	*P. lucens > E. nuttallii* = *V. spiralis*
		Species	2, 110	318.23	**<0.001**	
		Temp.* Species	2, 110	9.23	**<0.001**	
	N:Phenolics ratio	Temperature	1, 13	4.13	0.063	*E. nuttallii* = *V. spiralis* > *P. lucens*
		Species	2, 110	69.74	**<0.001**	
		Temp.* Species	2, 110	6.51	**0.002**	

*Means comparison among species was done by a Tukey post-hoc test after each linear mixed-effect model test. Temp. indicates the factor temperature*.

a *TIN means total dissolved inorganic nitrogen*.

b *RCR means plant relative consumption rate. Bold numbers indicate a significance of p < 0.05*.

Sediment porewater total inorganic nitrogen (TIN) and PO${}_{4}^{3-}$ concentrations decreased as temperature increased. The concentrations also differed among species, with a higher porewater nutrient content for *E. nuttallii* than for the other two species (Table 1, Figures 1E,F). During the experiment, alkalinity decreased from 1.5 to 1.0 meq L^−1^ (Figure S1d). Nutrients (N and P) were limiting in the water layer (almost 0 after the first 2 weeks, Figures S1f–i), but not limited in the sediment (Figures 1E,F).

### Plant Traits

**Figure 2:**
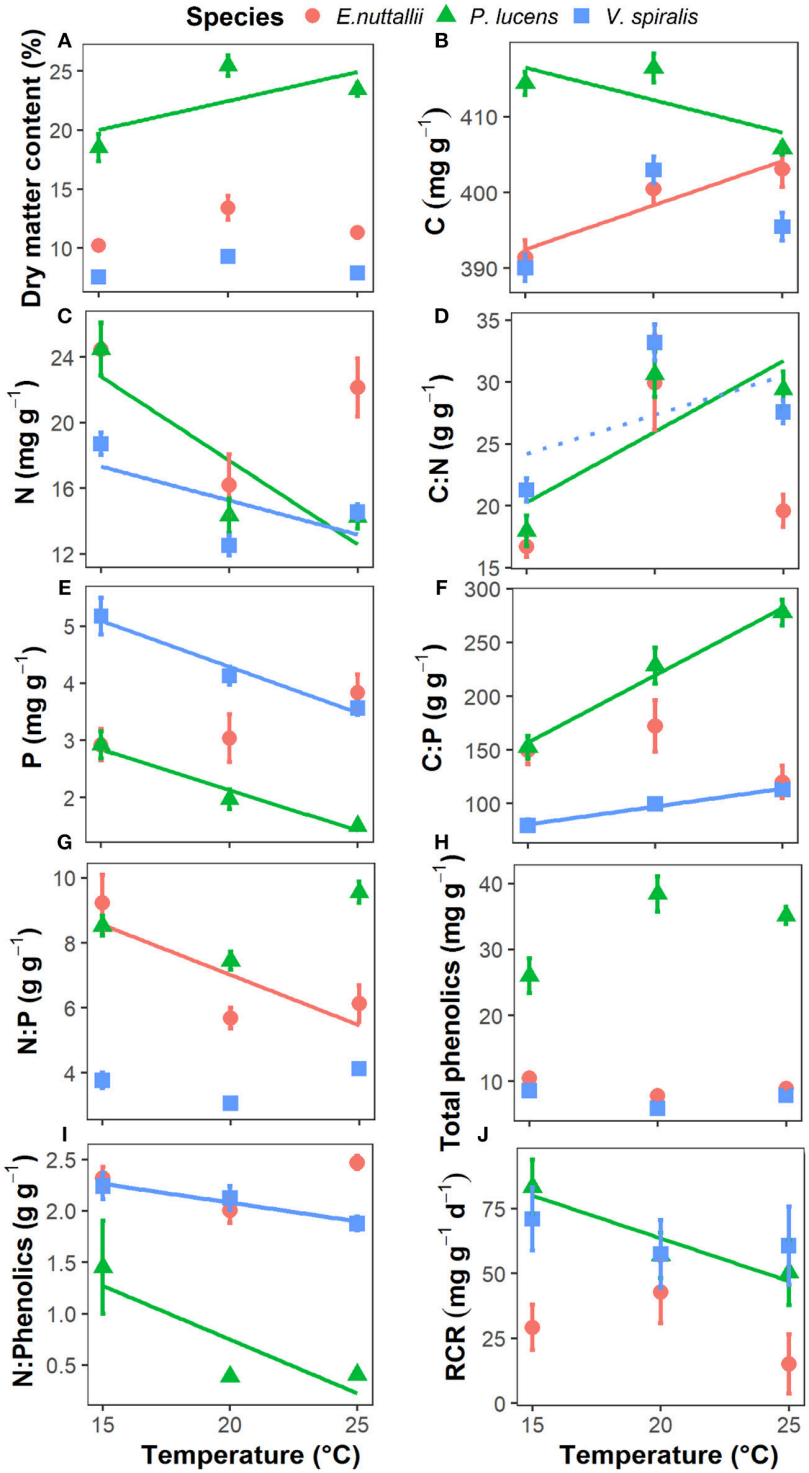
Response of aquatic plant traits and palatability to rising temperature in the three aquatic plants. **(A)** plant dry matter content, **(B)** plant C content, **(C)** plant N content, **(D)** plant C:N ratio, **(E)** plant P content, **(F)** plant C:P ratio, **(G)** plant N:P ratio, **(H)** plant total phenolics concentration, **(I)** plant N:Phenolics ratio, and **(J)** plant palatability indicates plant relative consumption rate, RCR. Point with bar represents mean ± SE (*n* = 15 for each point). Temperature effects on the parameters of each species are indicated by regression lines. A solid line indicates *p* < 0.05, a dotted line indicates 0.05 < *p* < 0.1, and without line indicates *p* > 0.1.

Plant stoichiometry showed a species-specific response to rising temperatures (Table 1, Figure 2). Plant C content decreased in *P. lucens*, remained unaltered in *V. spiralis*, and decreased in *E. nuttallii* as temperature increased (Table 1, Figure 2B). Temperature had an effect on both plant N and P content, which showed a significant decrease with rising temperature for *P. lucens* and *V. spiralis*, though there was no significant influence on *E. nuttallii* (Figures 2C,E). The plant C:N ratio significantly increased for *P. lucens* but not for the other species (Figure 2D). The plant C:P ratio significantly increased with rising temperature in both *P. lucens* and *V. spiralis*, whereas there were no effects on *E. nuttallii* (Figure 2F). The plant N:P ratio significantly decreased in *E. nuttallii* but not in the other species, whereas *V. spiralis* had the lowest N:P ratio (Table 1, Figure 2G). The plant N content was the highest and C:N ratio was the lowest in *E. nuttallii*, whereas the plant P content was the highest and C:P ratio was the lowest in *V. spiralis* (Table 1).

**Figure 3:**
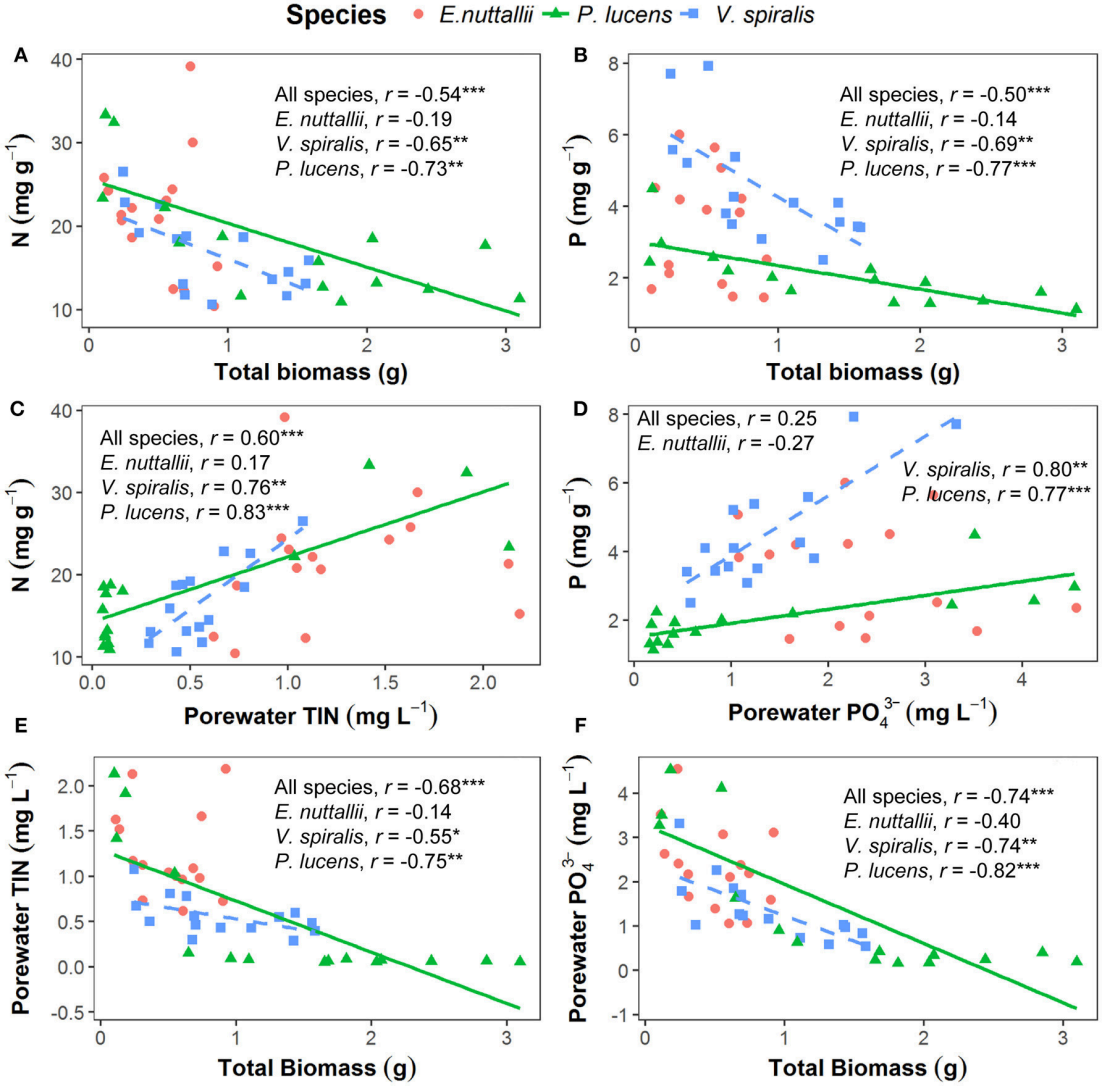
Pearson's correlations of plant total biomass, plant nutrient contents and porewater nutrient concentrations for the three tested species. Each species has 15 data points from every temperature treatment, in total 45 points for each panel. Lines in the graph indicate that factors are significantly correlated at the species level (*p* < 0.05). **(A)** N content in the plants in relation to the total plant biomass. **(B)** P content in the plants in relation to the total plant biomass. **(C)** N content in the plants in relation to the total inorganic nitrogen concentration in the porewater. **(D)** P content in the plants in relation to the phosphorous concentration in the porewater. **(E)** Total plant biomass in relation to the total inorganic nitrogen concentration in the porewater. **(F)** Total plant biomass in relation to the phosphorous concentration in the porewater. ^*^p < 0.05, ^**^p < 0.01, ^***^p < 0.001.

In *P. lucens* and *V. spiralis*, we found that foliar N and P content were negatively correlated with plant total biomass (Figures 3A,B) and were positively correlated with sediment porewater TIN concentration (Figure 3C) and PO${}_{4}^{3-}$ concentration (Figure 3D), respectively. The sediment porewater nutrient concentrations were negatively correlated with plant total biomass (Figures 3E,F).

Other plant traits also showed species-specific responses (Table 1, Figure 2). Plant dry matter content significantly increased with rising temperature in *P. lucens*, but not in the other species. *P. lucens* had the highest dry matter content (Table 1, Figure 2A). For plant total phenolics, there was no temperature effects for any of the species, whereas *P. lucens* had the highest total phenolics content (Figure 2H). In contrast, for the plant N:Phenolics ratio, both *P. lucens* and *V. spiralis* showed a significant decrease with rising temperature, whereas no significant influence was found on *E. nuttallii*. *P. lucens* had the lowest N:Phenolics ratio (Table 1, Figure 2I).

There were several general correlations among chemical plant traits in all three species (Table 2). In all species, dry matter content correlated negatively with N content, and positively with plant C:N ratio. N content and P content were positively correlated with each other in all species. Most chemical plant traits were correlated with each other within the aquatic plant species, but there were differences between the species as to the strength and direction of the correlations (Table 2).

**Table 2:** Pearson's correlation coefficients among all the investigated plant quality traits for all three species pooled (*n* = 45), and for each species separately (*n* = 15).

		**RCR^**a**^**	**Dry matter^**b**^**	**C**	**N**	**P**	**C: N**	**C: P**	**N: P**	**Phenolics**
All species	RCR^a^	1								
	Dry matter^b^	0.06	1							
	C	0.18	0.75^***^	1						
	N	0.04	**-0.25**	−0.09	1					
	P	0.04	−0.81^***^	−0.51^***^	0.32^*^	1				
	C: N	0.03	0.33^*^	0.26	-0.91^***^	-0.41^**^	1			
	C: P	−0.03	0.86^***^	0.49^***^	-0.37^*^	-0.93^***^	0.48^***^	1		
	N: P	−0.02	0.61^***^	0.34^*^	0.38^**^	−0.71^***^	−0.28	0.67^***^	1	
	Phenolics	0.10	0.89^***^	0.68^***^	**-0.10**	0.64^***^	**0.11**	0.69^***^	0.58^***^	1
	N: Phenolics	−0.05	−0.87^***^	−0.59^***^	0.48^***^	0.75^***^	−0.45^**^	-0.79^***^	−0.41^**^	−0.86^***^
*E. nuttallii*	RCR^a^	1								
	Dry matter^b^	0.30	1							
	C	0.10	0.42	1						
	N	−0.17	−0.85^***^	−0.09	1					
	P	−0.50	−0.70^**^	0.12	0.61^*^	1				
	C: N	0.34	0.93^***^	0.30	−0.88^***^	−0.64^*^	1			
	C: P	0.52^*^	0.81^***^	0.08	−0.68^**^	−0.92^***^	0.81^***^	1		
	N: P	0.27	−0.23	−0.34	0.42	−0.43	−0.33	0.26	1
	Phenolics	−0.02	−0.80^***^	−0.22	0.92^***^	0.39	−0.81^***^	−0.49	0.61^*^	1
	N: Phenolics	−0.44	−0.74^**^	0.02	0.82^***^	0.77^***^	−0.82^***^	−0.81^***^	0.06	0.55^*^
*V. spiralis*	RCR^a^	1								
	Dry matter^b^	−0.08	1							
	C	0.05	0.81^***^	1						
	N	0.27	−0.83^***^	−0.79^***^	1					
	P	0.21	−0.42	−0.54^*^	0.73^**^	1				
	C: N	−0.22	0.89^***^	0.83^***^	−0.98^***^	−0.65^**^	1			
	C: P	−0.23	0.38	0.53^*^	−0.69^**^	−0.96^***^	0.62^*^	1		
	N: P	0.02	−0.66^**^	−0.43	0.48	−0.22	−0.57^*^	0.29	1
	Phenolics	0.14	−0.71^**^	−0.70^**^	0.80^***^	0.26	−0.81^***^	−0.25	0.78^***^	1
	N: Phenolics	0.22	−0.30	−0.23	0.41	0.78^***^	−0.38	−0.73^**^	−0.35	−0.20
*P. lucens*	RCR^a^	1								
	Dry matter^b^	−0.69^**^	1							
	C	0.03	0.17	1						
	N	0.82^***^	−0.92^***^	0.08	1					
	P	0.79^***^	−0.79^***^	0.38	0.92^***^	1				
	C: N	−0.77^***^	0.87^***^	−0.06	−0.97^***^	−0.86^***^	1			
	C: P	−0.76^***^	0.71^**^	−0.42	−0.88^***^	−0.96^***^	0.87^***^	1		
	N: P	0.06	−0.30	−0.72^**^	0.19	−0.19	−0.27	0.24	1
	Phenolics	−0.57^*^	0.74^**^	0.35	−0.65^**^	−0.46	0.59^*^	0.45	−0.31	1
	N: Phenolics	0.85^***^	−0.89^***^	−0.06	0.90^***^	0.81^***^	−0.79^***^	−0.71^**^	0.20	−0.74^**^

*The directions of the significant correlations in all three species that are the same or different are indicated as bold green or bold red in the table, respectively*.

a *Plant relative consumption rate*.

b *Plant dry matter content*.

* *p < 0.05*,

** *p < 0.01*,

*** *p < 0.001*.

### Plant Palatability

Plant palatability (expressed as the relative consumption rate by the snails, RCR), generally showed a decreasing trend with increasing temperature (*p* = 0.067) (Table 1, Figure 2J), but differed among species (*p* < 0.001) (Table 1), *P. lucens* and *V. spiralis* were more palatable than *E. nuttallii*. On a species level, the palatability of *P. lucens* decreased 39.8% with rising temperature from 15 to 25°C. Palatability was not related to any of the measured plant traits when all species were pooled. Intraspecifically, in *P. lucens*, palatability was negatively correlated with dry matter content, C:N, and C:P ratio and total phenolics and it correlated positively with N and P content and the N:Phenolic ratio (Table 2). For *E. nuttallii*, only the plant C:P ratio correlated positively with palatability, while for *V. spiralis*, none of the measured plant traits correlated significantly with palatability (Table 2).

## Discussion

In this study, we tested the effects of water temperature on the growth, chemical plant traits and the resultant palatability of three submerged aquatic plants. Temperature rise significantly increased plant growth, increased tissue C: nutrient ratios and there was a trend toward lower palatability, but interestingly, some of these effects were species-specific.

### Plant Growth

Rising temperatures enhanced plant growth in our experiment, confirming our first hypothesis, which has also been previously observed in the laboratory (Barko and Smart, 1981; Madsen and Brix, 1997; Velthuis et al., 2017) and the field (Rooney and Kalff, 2000; Feuchtmayr et al., 2009). The optimum temperatures for photosynthesis for temperate submerged aquatic plants are usually located between 25 and 32°C (Barko et al., 1982; Santamaría and Van Vierssen, 1997; Pedersen et al., 2013), so our highest temperature of 25°C was close to optimal for growth. Periphyton growth can reduce light availability to aquatic plants and limit the growth of aquatic plants (Köhler et al., 2010). This can also partly explain lower growth of the plants at lower temperatures in our experiment, as there was more periphyton growth at lower temperatures (Figure S2). The growth of plants was probably not limited by carbon availability during the experiment, as the alkalinity was always above 1.0 meq L^−1^ (Vestergaard and Sand-Jensen, 2000a,b). In our study, the pH was above 8, which meant that the major carbon source was bicarbonate, and the species we used can all utilize bicarbonate as carbon source (Pedersen et al., 2013; Hussner et al., 2016). The plant species that we used can take up nutrients from both sediment and water (Carignan and Kalff, 1980; Barko et al., 1986; Rattray et al., 1991; Eugelink, 1998; Thiébaut, 2005). Nutrients were limited in the water during the experiment (Figure S1); there were much higher amounts of nutrients available in the sediment, hence this was the main source of nutrients for plant growth. It seemed that *P. lucens* had the best resource uptake strategy, as it rooted deep into the sediment, which we observed when washing the roots, and the nutrients in the sediment were depleted the fastest. *V. spiralis* developed roots only shallowly into the sediment, and *E. nuttallii* barely developed roots in the sediment, its roots were mainly in the water. *E. nuttallii* can take up nutrients from the water if these are available (Eugelink, 1998; Thiébaut, 2005). That could also be the reason that *E. nuttallii* formed the lowest biomass of the three species, as this species may have suffered from the nutrient limitations in this experiment. According to optimal partitioning theory, plants allocate more biomass to the roots when the available nutrients are lower in the sediment (Bloom et al., 1985). As we measured lower levels of nutrients in the sediment at higher temperatures in our experiment, and lower root:shoot ratios in *E. nuttallii* and *P. lucens*, it seems that at higher temperatures, these plants can utilize nutrients better to accumulate biomass, or these plants can grow faster leading to less nutrients being available. Indeed, as temperature increased, *E. nuttallii* and *P. lucens* showed a decrease in root:shoot ratio, which is consistent with the optimal partitioning theory.

### Plant Traits

Our results showed that higher temperature led to faster growth and lower nutrient availability, which in turn led to lower tissue nutrients in two of the three plant species (*P. lucens* and *V. spiralis*). The observed shifts in nutrient content and stoichiometry follow the temperature-plant physiological hypothesis (Reich and Oleksyn, 2004), which predicts that plant N and P content declines with increasing temperatures. At higher temperatures plants invest less nutrients per carbon for their metabolism and growth (Reich and Oleksyn, 2004; Zhang et al., 2016). This corresponds with our finding that there were lower levels of nutrients in the sediment at higher temperatures at which these plants can utilize nutrients better to accumulate biomass. We also found that there were strong negative correlations between macrophyte biomass and plant nutrient content and positive correlations between plant nutrient content and sediment porewater nutrient concentration. This means that there was a strong effect of nutrient dilution in plant tissue by increasing total biomass. This effect was not seen in *E. nuttallii*, which may have been less efficient in obtaining nutrients from the sediment and may have suffered nutrient limitation during the experiment. Previous studies argued that the critical nutrient contents for 95% maximum yield for *E. nuttallii* were *N* = 1.6% and *P* = 0.14% of their dry mass (Gerloff, 1975; Demars and Edwards, 2007), which are lower than the N and P contents in our experiment. However, *E. nuttallii* could still have suffered from nutrient limitation in our experiment if the critical nutrient contents were altered by changing temperature.

The tissue stoichiometry for *E. nuttallii*, and N content and C:N ratio for *P. lucens* and *V. spiralis* between 20 and 25°C seems to deviate from the general trend. This might have been caused by altered nutrient availability in the water layer at higher temperatures. Warming increases sediment respiration which probably increases the nutrient release from the sediment to the water (Liboriussen et al., 2011; Alsterberg et al., 2012; Zhang et al., 2012); this might result in higher nutrient availability at higher temperatures in the water layer (Ventura et al., 2008). These nutrients in the water could be taken up by aquatic plants, periphyton, and phytoplankton (van Donk and van De Bund, 2002). There was less periphyton at higher temperatures (Figure S2; possibly related to an increased grazing pressure by the periphyton grazing snails at higher temperatures), and more phytoplankton accumulated at higher temperatures. All in all, the rising temperature might have affected the nutrient availability in the water, and resulted in the differential responses of tissue stoichiometry in the aquatic plants.

Dry matter content has been assumed to be negatively correlated with plant nutrient content (Elger and Willby, 2003), which was true in all our three species. As temperature increased, plant nutrient content decreased, and then we can expect an increase in plant dry matter content. Rising temperature increased plant dry matter content in *P. lucens*, but not in the other two species.

There was no temperature effect on the total phenolics content, which is consistent with previous research on terrestrial plants (Jamieson et al., 2014). *P. lucens* had the highest total phenolics content among the three species, but was also preferred by *L. stagnalis*. This may have been due to the low total phenolic concentrations that we measured in our plants, even in *P. lucens*, compared to other aquatic plants species (Grutters et al., 2017). In the comparison among 40 aquatic plants species of Grutters et al. (2017), 36 species have a higher phenolic concentration than *P. lucens* in our study. Hence, the total phenolic concentration may have been too low to deter snail feeding, or *L. stagnalis* may be able to detoxify some toxic compounds (Gérard et al., 2005; Lance et al., 2006; Zurawell et al., 2007). Previous studies also showed that total phenolics could not adequately predict aquatic plant palatability (Elger and Lemoine, 2005; Boiché et al., 2011). Furthermore, the correlation between N content and total phenolics concentration showed different directions in the three plant species. This may also indicate that total phenolics can at best be considered a rough indicator of plant defense in aquatic plants (Gross and Bakker, 2012), whereas there are specific phenolic compounds that determine anti-herbivore defenses (Bidart-Bouzat and Imeh-Nathaniel, 2008; Harvey, 2015). However, the identity of these compounds is at present largely unknown in most freshwater plants.

### Plant Palatability

Because we observed the hypothesized changes in plant growth and in plant nutrient content and stoichiometry in two of our three tested plant species, we also expected that plant palatability would be reduced with increasing temperature. Indeed, aquatic plant palatability showed a decreasing trend as temperature increased, but this was at the species level, only significant in *P. lucens*. Also other studies which used different species found that warming either decreased marine plant palatability (Rodil et al., 2015), or had no effect (Poore et al., 2016). Therefore, we conclude that the effect of warming on plant palatability is to a certain extent species-specific, in our study depending on the plant species identity. In analogy, variation in the palatability of seaweeds across latitudes was recently found to vary with both plant and herbivore identity (Demko et al., 2017), and different generalist herbivores might respond differently to the same plant (Boiché et al., 2011). Here, it should be noted that we measured a plastic response of plants to temperature within a generation, whereas latitudinal gradients in plant traits and palatability are the result of selection pressures operating over generations. Similarly, the measured responses are short-term, whereas alterations in plant traits in response to climate change, including global warming, would be a slow process operating over generations.

Overall, in our study, plant palatability was significantly negatively correlated with plant dry matter content, C:nutrient ratio and total phenolics, and positively correlated with plant nutrient (N and P) content and N:Phenolics ratio in *P. lucens*, but not in the other two plant species. Hence, all hypothesized relationships between plant traits and palatability, based on the literature, were true for *P. lucens*. *P. lucens* also responded to temperature rise as we expected both in its growth, chemical traits and palatability and moreover, we can understand the responses, as they are coherent with each other. However, *P. lucens* is clearly not representative for all aquatic plants, as the other two tested species responded differently and less consistently in their plant growth, chemical traits and palatability relationships. Across a wide range of aquatic plant species palatability increases with decreasing dry matter content (Elger and Willby, 2003; Elger and Lemoine, 2005), and increasing N:phenolics ratio (Grutters et al., 2017), and among different functional plant groups, consumption rates increased with N content (Cebrian and Lartigue, 2004) and decreased with C:nutrient ratio (Elser et al., 2000; Bakker et al., 2016; Grutters et al., 2016). Possibly, the measured plant traits might be better in predicting plant palatability on an interspecies level, instead of intraspecifically.

### Implications for the Aquatic Ecosystem

The plant species tested differed strongly in resource uptake and growth, which may give some species competitive advantages over other species in warming ecosystems. Consequently, warming might alter the aquatic plant community composition (McKee et al., 2002; Zhang et al., 2015; Li et al., 2017). Similarly, under current global warming trends, the stoichiometric mismatch with higher trophic levels may enlarge with an increasing carbon:nutrient ratio in some plant species. As a consequence, the palatability difference between plant species may change, which may lead to a different pressure from herbivores on some species as compared to others, which may also change the aquatic plant community composition and abundance (Schiel et al., 2004; Harley et al., 2012).

Water temperature can affect aquatic plant-herbivore interactions in aquatic ecosystems by (1) affecting plant palatability or (2) affecting grazing rate of ectothermic animals (O'Connor, 2009). As ectotherm animals ingest more food with increasing temperatures (Zhang et al., 2018), and the consumption rates of animals increase faster than the plant growth rates with rising temperature (West and Post, 2016; Schaum et al., 2018), this can lead to enhanced top-down control on aquatic plants (O'Connor, 2009). However, our data show that aquatic plant palatability and stoichiometry decrease in some species with rising temperature, suggesting that plant quality may decrease with increasing temperatures. The question is whether plants remain a viable food source to sustain the ectotherm consumer population. Our study demonstrates the need to explore the effects of temperature on aquatic plant-consumer interactions at an ecosystem level.

## Author Contributions

PZ, JX, and EB came up with the research question and designed the study approach. PZ, BG, AP, RvdB, and EB designed and conducted the experiment. PZ, BG, and CvL performed the data analyses and statistics. PZ, BG, CvL, JX, AP, RvdB, and EB wrote the paper.

### Conflict of Interest Statement

The authors declare that the research was conducted in the absence of any commercial or financial relationships that could be construed as a potential conflict of interest. The handling Editor declared a past co-authorship with EB.
