# Supplementary material for: Effects of Rising Temperature on the Growth, Stoichiometry, and Palatability of Aquatic Plants
Source: Front Plant Sci. 2019 Jan 8;9:1947. doi: 10.3389/fpls.2018.01947 (PMC6331454; doi:10.3389/fpls.2018.01947)
Supplement: Supplementary file 1 [file Data_Sheet_1.docx]

**
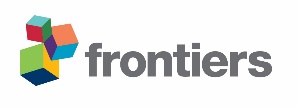
**

***Supplementary Material***

Article title: Effects of rising temperature on the growth, stoichiometry and palatability of aquatic plants

Authors: Peiyu Zhang, Bart M.C. Grutters, Casper H. A. van Leeuwen, Jun Xu, Antonella Petruzzella, Reinier F. van den Berg, Elisabeth S. Bakker

The following Supporting Information is available for this article:

**Fig. S1** Water quality measurements during 16 weeks of the aquatic plants growth experiment, at three different water temperatures.

**Fig. S2** Final periphyton biomass that developed at the three different temperature treatments.

**Table S1** Model selection based on AICc values.


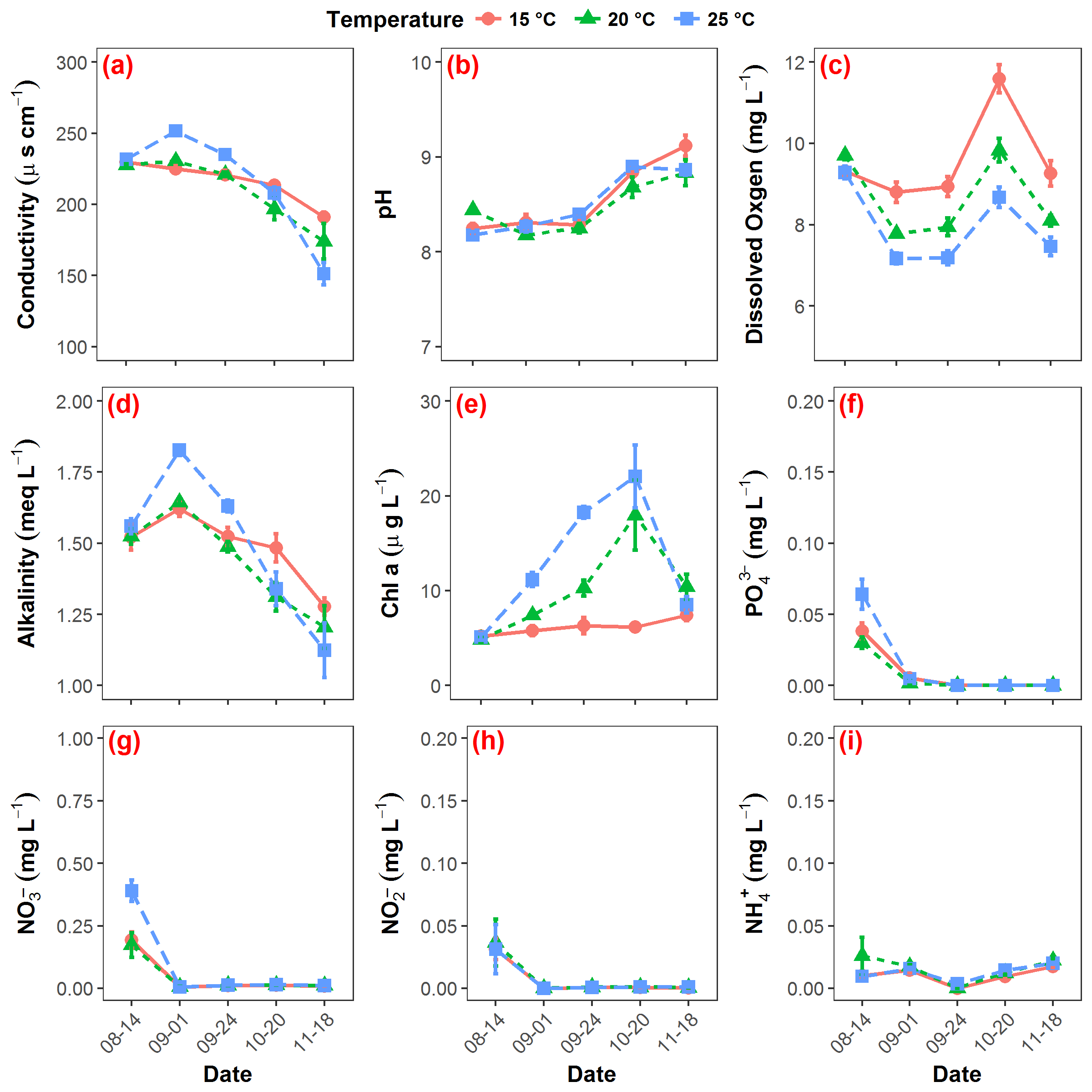


**Fig. S1** Water quality measurements during 16 weeks of the aquatic plants growth experiment, at three different water temperatures. (a) Conductivity; (b) pH; (c) Dissolved Oxygen; (d) Alkalinity; (e) Chlorophyll a; (f) PO_4_^3-^; (g) NO_3_^-^; (h) NO_2_^-^; (i) NH_4_^+^. Error bars are standard deviations.


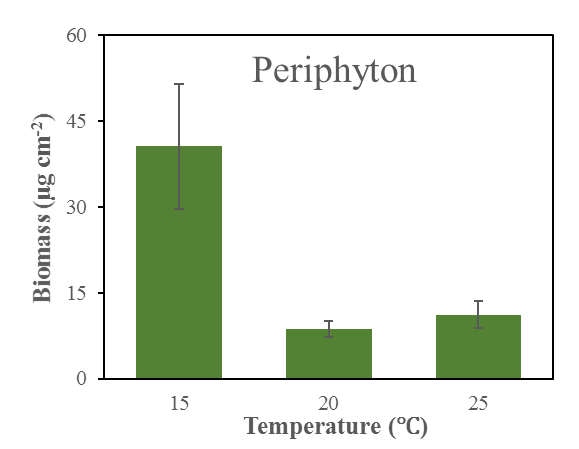


**Fig. S2** Final periphyton biomass that developed at the three different temperature treatments. One-way mixed effect model (with temperature as a continuous predictor and aquarium as a random factor) indicates a significant (*F*_1,13_ = 7.42, *p* = 0.017) decrease of periphyton biomass with rising temperature. Error bars indicate standard errors.

**Table S1** Model selection based on AICc values. Fixed effects were fitted by backward selection based upon AICc values calculated using ML estimation. Aquarium is the random factor for all the models. Models with the lowest AICc value and those within 2.0 ΔAICc of this model are indicated in bold. An X indicates the factors included in each model.

| **Category** | **Parameters** | **Model** | **Plant species × Temperature** | **Plant species** | **Temperature** | **ΔAICc** |
| --- | --- | --- | --- | --- | --- | --- |
| **Plant growth** | Shoot | **1** | **X** | **X** | **X** | **Δ 0.0** |
|  |  | 2 |  | X | X | Δ +64.72 |
|  |  | 3 |  | X |  | Δ +105.89 |
|  |  | 4 |  |  | X | Δ +135.02 |
|  | Root | **1** | **X** | **X** | **X** | **Δ 0.0** |
|  |  | 2 |  | X | X | Δ +31.70 |
|  |  | 3 |  | X |  | Δ +55.97 |
|  |  | 4 |  |  | X | Δ +145.44 |
|  | Growth rate | **1** | **X** | **X** | **X** | **Δ 0.0** |
|  |  | 2 |  | X | X | Δ +11.88 |
|  |  | 3 |  | X |  | Δ +32.28 |
|  |  | 4 |  |  | X | Δ +251.93 |
|  | Root:Shoot | **1** | **X** | **X** | **X** | **Δ 0.0** |
|  |  | 2 |  | X | X | Δ +35.78 |
|  |  | 3 |  | X |  | Δ +38.08 |
|  |  | 4 |  |  | X | Δ +249.26 |
| **Porewater nutrients** | TIN | **1** | **X** | **X** | **X** | **Δ +1.40** |
|  |  | 2 |  | X | X | Δ 0.0 |
|  |  | 3 |  | X |  | Δ +1.07 |
|  |  | 4 |  |  | X | Δ +13.46 |
|  | PO_4_^3-^ | **1** | **X** | **X** | **X** | **Δ 0.0** |
|  |  | 2 |  | X | X | Δ +0.76 |
|  |  | 3 |  | X |  | Δ +10.51 |
|  |  | 4 |  |  | X | Δ +8.56 |
| **Palatability** | RCR | 1 | X | X | X | Δ +3.45 |
|  |  | **2** |  | **X** | **X** | **Δ 0.0** |
|  |  | 3 |  | X |  | Δ +1.77 |
|  |  | 4 |  |  | X | Δ +11.93 |
| **Traits** | Dry matter | **1** | **X** | **X** | **X** | **Δ 0.0** |
|  |  | 2 |  | X | X | Δ +12.40 |
|  |  | 3 |  | X |  | Δ +12.98 |
|  |  | 4 |  |  | X | Δ +247.96 |
|  | C | **1** | **X** | **X** | **X** | **Δ 0.0** |
|  |  | 2 |  | X | X | Δ +25.70 |
|  |  | 3 |  | X |  | Δ +24.59 |
|  |  | 4 |  |  | X | Δ +97.24 |
|  | N | **1** | **X** | **X** | **X** | **Δ 0.0** |
|  |  | 2 |  | X | X | Δ +8.83 |
|  |  | 3 |  | X |  | Δ +12.05 |
|  |  | 4 |  |  | X | Δ +32.97 |
|  | P | **1** | **X** | **X** | **X** | **Δ 0.0** |
|  |  | 2 |  | X | X | Δ +26.78 |
|  |  | 3 |  | X |  | Δ +29.57 |
|  |  | 4 |  |  | X | Δ +90.80 |
|  | C:N | **1** | **X** | **X** | **X** | **Δ 0.0** |
|  |  | 2 |  | X | X | Δ +6.67 |
|  |  | 3 |  | X |  | Δ +8.98 |
|  |  | 4 |  |  | X | Δ +30.52 |
|  | C:P | **1** | **X** | **X** | **X** | **Δ 0.0** |
|  |  | 2 |  | X | X | Δ +29.11 |
|  |  | 3 |  | X |  | Δ +33.92 |
|  |  | 4 |  |  | X | Δ +100.38 |
|  | N:P | **1** | **X** | **X** | **X** | **Δ 0.0** |
|  |  | 2 |  | X | X | Δ +22.54 |
|  |  | 3 |  | X |  | Δ +21.23 |
|  |  | 4 |  |  | X | Δ +131.15 |
|  | Total phenolics | **1** | **X** | **X** | **X** | **Δ 0.0** |
|  |  | 2 |  | X | X | Δ +13.13 |
|  |  | 3 |  | X |  | Δ +13.40 |
|  |  | 4 |  |  | X | Δ +223.15 |
|  | N:Phenolics | **1** | **X** | **X** | **X** | **Δ 0.0** |
|  |  | 2 |  | X | X | Δ +8.21 |
|  |  | 3 |  | X |  | Δ +10.62 |
|  |  | 4 |  |  | X | Δ +91.52 |
